# Purcell Effect in Epsilon-Near-Zero Microcavities

**DOI:** 10.1021/acsomega.5c07448

**Published:** 2025-09-20

**Authors:** Ali Panahpour, Jussi Kelavuori, Mikko Huttunen

**Affiliations:** Photonics Laboratory, Physics Unit, 528748 Tampere University, FI-33014 Tampere, Finland

## Abstract

Epsilon-near-zero (ENZ) photonics offers a compelling platform for integrated photonic systems, enabling a range of novel and extraordinary functionalities. However, the practical deployment of ENZ-based devices is constrained by high material losses and severe impedance mismatch, which are detrimental to applications requiring coherent light manipulation and efficient light-matter interaction. To address these challenges, we demonstrate that all-dielectric Bragg-reflection microcavities operated near their cutoff frequency, offer an ultralow-loss platform for enhancing light-matter interaction and exploring emission processes in the ENZ regime. While Bragg cavities are well-established, their potential as ENZ resonant microcavities remains largely unexplored. We investigate the Purcell effect and quality factor in these structures, comparing their performance with those of the perfect-electric-conductor and metallic counterparts. Through analytical derivations based on Fermi’s golden rule and field quantization in lossless dispersive media, we establish scaling laws that distinguish these ENZ cavities from conventional resonators. Frequency domain simulations validate our counterintuitive findings, demonstrating that in all-dielectric ENZ Bragg-reflection microcavities, the Purcell and quality factors scale as *L*/λ_0_ and (*L*/λ_0_)^3^, respectively, where *L* is the cavity length and λ_0_ is the resonance wavelength. Our results offer key insights into the design of ENZ-based photonic systems, paving the way for enhanced light-matter interactions in nonlinear optics and quantum photonics.

## Graphical abstract

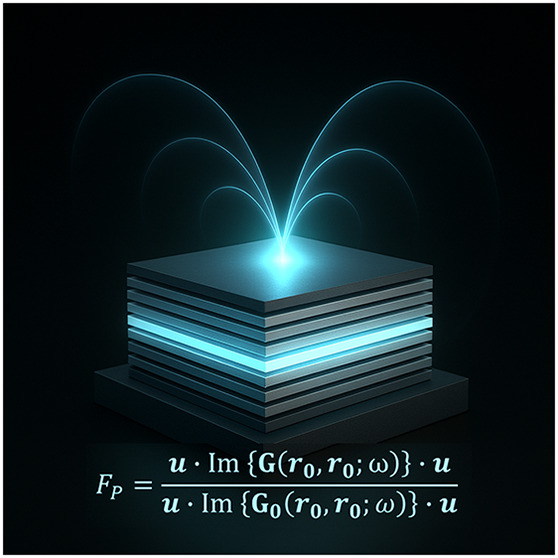

## 1 Introduction

Epsilon-near-zero (ENZ) photonics provides a novel platform for developing new types of integrated photonic devices with unconventional functionalities.^1–10^ This is due to variety of extraordinary effects, offered by ENZ materials and structures, such as energy squeezing,^11^ tunneling through subwavelength channels and bends,^12^ enhancement of optical nonlinearities,^5,13^ efficient and coherent coupling of emitters for quantum entanglement^14,15^ or quantum networks^16^ and boosting of the Purcell factor (PF) in radiative systems.^8,17–19^ Conventional ENZ materials or nanostructures have been mainly based on conducting oxide or nitride materials as well as metamaterials consisting of metal–dielectric or semiconductor–dielectric components.^3,4^ Metallic waveguides, near their cutoff frequencies have also been utilized to emulate the properties of an ENZ medium.^18,20^

Despite their promising potential, the performance of ENZ-based devices is strongly influenced by material losses, which can be either advantageous or detrimental depending on the specific application and the nature of the light-matter interaction. In certain cases, absorption is not only desirable but essential, such as in ENZ-based perfect absorbers for thin-film sensing,^21^ or in the enhancement of intensity-dependent refractive index in ENZ materials like doped oxides and nitrides.^22^ In contrast, in many other contexts, optical losses pose a significant challenge. Applications such as wave tunneling, wavefront shaping, electro-optic modulation, all-optical switching, and quantum emitter coupling demand low-loss ENZ behavior to preserve coherence, facilitate efficient energy transfer, and minimize undesired dissipation.^5,23^ In these scenarios, reducing absorption is critical for maintaining device performance and ensuring high fidelity in both quantum and classical photonic systems.

A key metric for evaluating the absorption in ENZ media is the figure of merit (FoM), defined as the ratio of the real to the imaginary parts of the complex permittivity, FoM = *ε*_r_/*ε*_i_. A high-FoM ENZ medium is characterized by the condition *ε*_i_ ≪ *ε*_r_ ≪ 1, corresponding to a low-loss regime that is essential for applications involving coherent light manipulation or transmission. In this limit, the FoM also closely approximates the ratio of the real to imaginary components of the complex refractive index, given by FoM = (*n* ^2^ – κ^2^)/(2*nκ*) ≈ *n*/2κ, since *ε*_i_ ≪ *ε*_r_ ≪ 1 implies κ ≪*n* ≪1.

The high-FoM condition delineates the validity domain of our results, as our theoretical framework relies on field quantization in a dispersive yet lossless medium. In fact, our results apply exactly to perfect electric conductor (PEC) cavities, which are idealized structures with no material losses, and approximately to realistic high-FoM microcavities operating in the ENZ regime. As discussed in Section 3.2, the high-FoM condition limits our analysis to Bragg-reflection (BR) cavity sizes below a few hundred micrometers, depending on the level of material losses within the cavity. We therefore refer to these structures as ENZ Bragg *microcavities* throughout this article.

One of the key applications of low-loss ENZ structures is the enhancement of the PF in radiative processes. Recent studies have shown that among the various subcategories of near-zero-index (NZI) media, the PF diverges in one-dimensional (in wavenumber (*k*)-space) ENZ structures, such as metallic waveguides, due to a substantial increase in the electromagnetic density of states.^24^ This ENZ-induced PF enhancement has also been experimentally demonstrated in rectangular metallic waveguides.^25,26^ However, in practical implementations, waveguides have finite lengths and therefore exhibit partial reflections at their ports, caused by impedance or effective-index mismatch with the surrounding medium. This reflection effect is especially pronounced in the ENZ regime, where the index mismatch is maximized (see Supporting Information (SI), Section 1). Therefore, a finite-length ENZ waveguide can behave as a resonator, with feedback provided by the port reflections. As a result, the theoretical models describing radiation in ideal, infinitely long ENZ waveguides do not fully capture the PF enhancement in realistic structures. A more accurate description must therefore extend the analysis to include ENZ cavities. In this work, in addition to developing such an extension, we propose and analyze BR microcavities operating near their cutoff frequency as a low-loss, all-dielectric ENZ platform suitable for applications that require ENZ behavior with minimal absorption. This is particularly important because many experimental demonstrations of the ENZ effect, both in linear and nonlinear systems^25–27^ are significantly constrained by intrinsic material losses.^28–30^ These losses hinder the full realization of Purcell enhancement in radiative processes, limiting the performance and applicability of ENZ-based devices.

Here, the PF is investigated in high-FoM ENZ waveguides and cavities, beginning with ideally lossless PEC structures as reference systems for comparison with their metallic and all-dielectric counterparts. Although perfectly lossless ENZ waveguides and cavities do not exist in practice, all-dielectric planar BR waveguides (BRWs)^31^ operating near cutoff can exhibit low-loss ENZ behavior, featuring strong dispersion and a high group index (*n*_g_) quite similar to PEC waveguides, as demonstrated in Section 3. This enhancement of *n*_g_ near cutoff has also been studied previously in the context of slow-light phenomena.^32,33^ Earlier work has further shown the potential to control the intensity, rate, or angular distribution of spontaneous emission using BR cavities.^34–36^ These studies were primarily based on the Fermi’s golden rule or first-order perturbation theory applied to multimode cavities with virtually infinite lateral dimensions. Here, we study the PF and quality factor (*Q*) in single-mode near-cutoff BR cavities, with laterally confined dimensions (Figure 1a), where the cavity mode frequency is set by the finite size of the structure.^37^ Our theoretical framework is based on the Fermi’s golden rule and field quantization in lossless dispersive media. The results are expressed in terms of effective refractive index of the cavity, which holds particular relevance in the context of ENZ/NZI structures. We also derive scaling laws for the PF and *Q* of such ENZ structures, revealing a counterintuitive trend of enhancement of PF by increasing the size of cavity.

**Figure 1:**
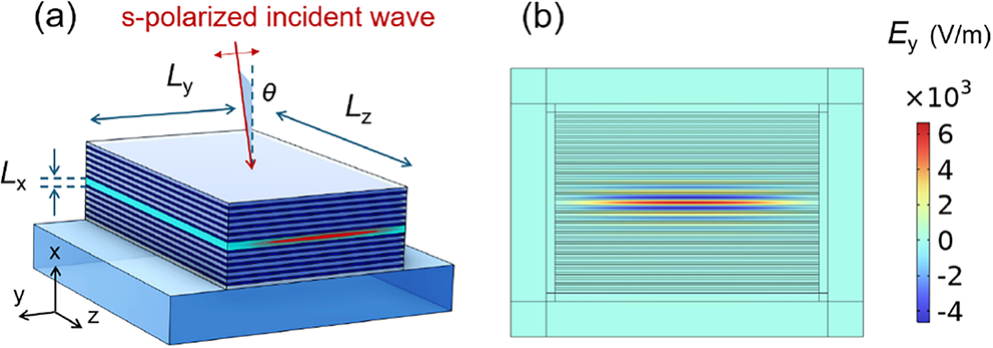
(a) Schematic view of a BR microcavity and (b) its transverse cross-section view, showing the *y*-component of electric field, corresponding to a dominant ENZ mode of the waveguide, computed by 2D mode-analysis simulation.

To place our results in context, we briefly revisit the conventional PF expression for resonant cavities. The perturbative effect of a structured vacuum on radiative processes, known as the Purcell effect, was originally demonstrated by placing an emitter inside a cavity.^38^ When the emitter’s transition frequency is tuned to the resonant mode of a *nondispersive* cavity, the spontaneous emission rate is enhanced by the factor

1 $$F_{P}=\frac{6}{\pi^{2}}{\left(\frac{\lambda }{2n_{c}}\right)}^{3}\frac{Q}{V_{m}}$$

where λ is the photon wavelength in vacuum, *n*_*c*_ is the refractive index of medium inside the cavity, *Q* is the quality factor of the cavity and *V*_*m*_ is the mode volume. Traditional approaches to maximizing the PF are mainly based on three different classes of photonic structures: ultrahigh-*Q* (∼10^8^) dielectric microcavities with *V*_*m*_ much larger than (λ/2*n*_*c*_)^3^,^39^ moderate *Q* (∼10^4^) photonic crystal nanocavities with mode volumes on the order of (λ/2*n*_*c*_)^3^,^40^ and finally plasmonic nanoresonators with extremely small *V*_*m*_ but relatively low *Q* (≤100). Strongly dispersive cavities represent an alternative route to enhance both *Q* and PF.^41–43^ However, eq 1 is valid only for nondispersive systems and therefore it does not accurately capture the Purcell enhancement in dispersive ENZ cavities.

Due to the inherently strong dispersion of high-FoM ENZ media, resonant ENZ structures are expected to exhibit significantly enhanced *Q* and Purcell effect. Moreover, the resonance of the cavity near the cutoff frequency of the structure can mitigate the challenge of weak coupling of light with ENZ media due to impedance mismatch. In nonresonant ENZ structures, this Impedance mismatch leads to strong reflection from interfaces and suppressed light-matter interaction. However, it can be harnessed by exploiting the ENZ interfaces as reflective boundaries, providing optical feedback and forming a Fabry–Pérot resonant cavity that enhances light-mater interaction. Furthermore, the elongation of wavelength within the ENZ region serves to alleviate scattering-based losses arising from structural imperfections,^44^ and potentially can improve mode overlap and phase matching in nonlinear processes.^13,45^

## 2 Analytical Formulation of PF in ENZ Microcavities

To study the Purcell effect in ENZ structures in the weak interaction regime, we apply Fermi’s golden rule giving the spontaneous emission rate (SER) of a quantum emitter

2 $$\Gamma =\frac{2\pi }{\hbar^{2}}{\left|M_{12}\right|}^{2}\rho \left(r_{0},\omega \right)$$

in terms of transition matrix element *M*_12_ and optical local density of states (LDOS), ρ­(**r**_0_, ω) at the position of the quantum emitter, **r**_0_. Applying the quantization procedure for a homogeneous and lossless dispersive medium,^24,46^ the transition matrix element is given by

3 $${\left|M_{12}\right|}^{2}=\frac{\hbar \omega }{2\varepsilon_{0}V_{m}}\frac{{\left|p\right|}^{2}}{n\left(\omega \right)n_{g}\left(\omega \right)}$$

with *n*, *n*_g_, *V*_*m*_ and **p** being the (real part of) medium phase index, group index, mode volume and emitter’s dipole moment, respectively. A straightforward method to realize low-dimensional ENZ structures is to use metallic waveguides with planar or rectangular geometries (Figure 3b), operating near their cutoff frequencies.^24^ In the case of a one-dimensional rectangular waveguide with transverse dimensions of *L*_*x*_ and *L*_*y*_, and with idealized PEC walls, the LDOS per unit frequency at the center of the waveguide for an arbitrary quantization length of *L*_*z*_ along *z*-axis is given by ρ­(**r**_0_, ω) = (*L*_*z*_/π*c*)*n*_g_(ω).^47^ Thus, by using eqs 2 and 3, the SER of an emitter in the waveguide is given by

4 $$\Gamma^{W}=\frac{|p{|}^{2}}{\varepsilon_{0}\hbar A_{m}}\frac{\omega }{c}\frac{1}{n}$$

where *A*_*m*_ = (∫*dx*∫*dy* |**E**_*m*_(*x*, *y*)|^2^)/|**E**_*m*_(**r**_peak_)|^2^ denotes the effective mode area of the waveguide, assuming a homogeneous medium with spatially invariant permittivity inside the cavity.^47^ The integration is performed over the squared electric field of the guided mode across the waveguide cross section and is normalized to its maximum value within the waveguide. For the dominant mode, this yields *A*_*m*_ = 0.5 *L*_*x*_ *L*_*y*_, as the field is uniform along one transverse direction and varies sinusoidally along the other. By normalizing eq 4 to the SER in free space, the PF turns into

5 $$F_{P}^{W}=\frac{3\pi }{A_{m}}\frac{c^{2}}{\omega^{2}}\frac{1}{n}$$

The effective index dispersion in the same PEC waveguide filled with a material of refractive index *n*_*c*_ is obtained by applying the expression for the propagation constant $\beta =\sqrt{k^{2}-k_{c}^{2}}$ , where *k* = *n*_*c*_ *k*_0_, *k*_0_ = 2π/λ is the free-space wavenumber and *k*_*c*_ = π/*L*_*x*_ is the cutoff wavenumber of the dominant TE_10_ mode.^48^ Using the relation β = *n*(λ)*k*_0_, for the propagation constant, the effective refractive index can be written as

6 $$n\left(\lambda \right)=n_{c}\sqrt{1-{\left(\frac{\lambda }{\lambda_{c}}\right)}^{2}}$$

where λ_*c*_ = 2π*n*_*c*_/*k*_*c*_ = 2*n*_*c*_ *L*_*x*_ is the cutoff wavelength of the guided mode.

To illustrate the implications of eq 5 for spontaneous emission enhancement and its divergence at the cutoff wavelength in the ideally lossless waveguide, the solid curve in Figure 2 shows the PF in a vacuum-filled PEC waveguide as a function of wavelength, calculated using the same equation. The cutoff wavelength and transverse dimensions of the waveguide are assumed to be λ_c_ = 1 μm, *L*_*x*_ = 0.5 μm and *L*_*y*_ = 1 μm, respectively.

**Figure 2:**
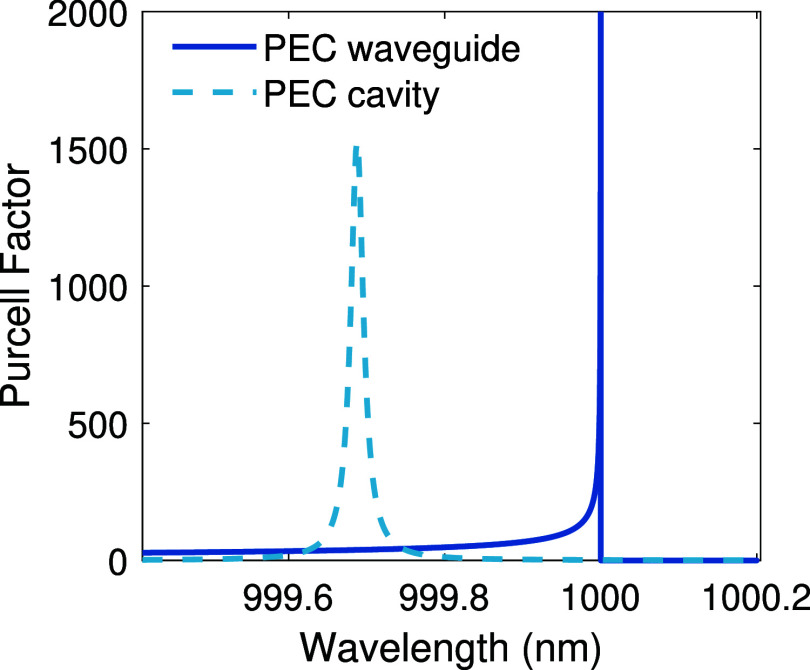
(Solid curve) calculated PF from eq 5 for a PEC waveguide with λ_*c*_ = 1 μm, *L*_*x*_ = 0.5 μm, *L*_*y*_ = 1 μm, and *A*_*m*_ = 0.5 *L*_*x*_ *L*_*y*_, corresponding to the dominant TE_10_ mode. (Dashed curve) the PF calculated from eq 12 for a PEC cavity with open ports, having the same transverse dimensions but a cavity length of *L*_*z*_ = 20 μm and the confinement factor of α = 0.25, corresponding to the TE_101_-like dominant mode.

According to eq 6, near the cutoff wavelength, the effective index *n* drops well below that of the surrounding medium. As a result, in a truncated waveguide, strong reflections occur at the waveguide terminations due to the index mismatch with the surroundings. Consequently, a finite-length waveguide can behave as a resonant cavity, supporting standing-wave modes that approximately satisfy the standard Fabry–Pérot phase accumulation condition (see SI, Section 1), leading to the resonance relation *n*(λ) *L*_*z*_ ≈ *m*λ/2, where *m* is an integer and *L*_*z*_ is the waveguide length. We focus on the Purcell effect in this structure, particularly at the wavelength of the resonant mode that is spectrally closest to the cutoff. The resonance wavelength is determined by the condition

7 $$n_{c}\sqrt{1-{\left(\frac{\lambda }{\lambda_{c}}\right)}^{2}}\approx \frac{m\lambda }{2L_{z}}$$

where *m* is the longitudinal mode number. For the dominant mode, we set *m* = 1 and use λ_*c*_ = 2*n*_*c*_ *L*_*x*_, leading to the following expression for the resonance wavelength

8 $$\lambda_{0}\approx \frac{2n_{c}L_{x}}{\sqrt{1+{\left(L_{x}/L_{z}\right)}^{2}}}$$

We assume that the cavity mode resonance is tuned to the emission frequency of a dipole emitter positioned at the center of the cavity. The LDOS at this point can then be written as a Lorentzian function ρ­(**r**_0_, ω) = (2δω/π)/(4­(ω – ω_0_)^2^ + δω^2^), reducing at resonance to ρ­(**r**_0_, ω_0_) = (2/π)­(*Q*/ω_0_). Here, *Q* = ω_0_/δω is the quality factor of the cavity with the resonance width of δω, which in terms of cavity finesse F , is given by $Q=FL_{z}\omega_{0}/\pi c$ . Since the cavity resonance is near the cutoff frequency, it exhibits strong dispersion, modifying the free spectral range to *c*/2*n*_g_ *L*_*z*_ and the dispersive cavity quality factor to *Q*_disp_ = *Qn*_g_.^41,42^ The LDOS is accordingly determined by ρ­(**r**_0_, ω_0_) = (2/π)­(*Q*/ω_0_)*n*_g_(ω_0_). Then applying eqs 2 and 3, the SER in the dispersive single-mode rectangular cavity is given by

9 $$\Gamma^{C}=\frac{2{\left|p\right|}^{2}}{\hbar \varepsilon_{0}V_{m}}\frac{Q}{n}$$

After normalizing to the SER in vacuum, the PF in terms of λ_0_ = 2π*c*/ω_0_ fulfills

10 $$F_{P}^{C}=\frac{3}{4\pi^{2}}\frac{\lambda_{0}^{3}}{V_{m}}\frac{Q}{n}$$

In eqs 1, 9, and 10, *V*_*m*_ denotes the effective mode volume, defined as^47^

11 $$V_{m}=\frac{\int_{V}\varepsilon \left(r\right){\left|E_{m}\left(r\right)\right|}^{2}dr}{\varepsilon \left(r_{\max}\right){\left|E_{m}\left(r_{\max}\right)\right|}^{2}}$$

in terms of the spatially varying permittivity *ε*(**r**) and the electric field distribution **E**_*m*_(**r**) of the cavity mode. The integration is carried out over the quantization volume *V*. Here, **r**_max_ denotes the position of the maximum electric field amplitude, and *ε*(**r**_max_) is the corresponding permittivity at that location. When the material inside the cavity is homogeneous, the mode volume simplifies to *V*_*m*_ = ∫_*V*_|**E**_*m*_(**r**)|^2^ d**r**/|**E**_*m*_(**r**_max_)|^2^. In this case, the mode volume can be expressed in terms of the cavity’s geometrical volume as *V*_*m*_ = *αV* where α = ⟨|**E**_*m*_(**r**)|^2^⟩/|**E**_*m*_(**r**_max_)|^2^ represents the ratio of the average to the peak electric field intensity within the cavity. It should be noted that the PF formulas given by relations (1) and (10) rely on the assumption that the emitter is optimally aligned with the cavity field polarization and precisely positioned at the point of maximum electric field, typically located at a field antinode.^49,50^

By comparing eq 10 with eq 1 for a cavity with fixed mode volume and reflectivity-limited *Q*, we find that by inclusion of dispersion, the derived PF exceeds the conventional estimate by a factor of *f* = *n*_*c*_^3^/*n*. For instance, with *n*_*c*_ = 1.5 and *n* = 0.01, the correction boosts the PF by 337.5 compared to the value given by eq 1.

### 2.1 Purcell Factor in PEC-Walled ENZ Cavities

To analyze the scaling behavior of eq 10 with respect to the NZI value *n*, we consider a PEC-walled ENZ cavity filled with material of refractive index *n*_*c*_ and modeled as an open-ended PEC waveguide of length *L*_*z*_ = λ_0_/2*n*, where the resonance wavelength λ_0_ is given by eq 8. The transverse dimensions are assumed to be *L*_*y*_ = λ_0_/*n*_*c*_ and *L*_*x*_ = λ_*c*_/2*n*_*c*_ (which approximates to λ_0_/2*n*_*c*_ for *L*_*z*_ ≫ *L*_*x*_). Using the relation $Q=\left(2n_{c}L_{z}/\lambda_{0}\right)F$ , we see from eq 10 that PF is proportional to F/n . In the absence of material losses, the cavity finesse F is determined by the reflectivity finesse, $F_{R}=\pi \sqrt{R}/\left(1-R\right)$ where *R* is the reflectivity at the cavity ports. For ENZ/NZI cavities surrounded by vacuum, and assuming the reflectivity *R* = ((*n* – 1)/(*n* + 1))^2^, the reflectivity-limited finesse is approximately $F_{R}\approx \pi /4n$ (SI, Section 1). Therefore, the PF and *Q* of the dispersive cavity are given by

12 $$F_{P}^{C}\approx \frac{3}{4\pi \alpha }\frac{n_{c}^{3}}{n^{2}}$$

13 $$Q_{\mathrm{disp}.}=Qn_{g}\approx \frac{\pi }{4}\frac{n_{c}}{n^{3}}$$

Then by using the fundamental cavity resonance condition *n* = λ_0_/2*L*_*z*_, we find *F*_P_^C^ ∝ (*L*_*z*_/λ_0_)^2^ and *Q*_disp._ ∝ (*L*_*z*_/λ_0_)^3^. These scaling laws are in contrast to the cases of ordinary microcavities, where PF is reduced by increasing the mode volume.

Equation 12 gives PF for an open-port PEC cavity at its resonance wavelength, which is assumed to coincide with the emission wavelength of an emitter placed at the center of the cavity. To show the spectral profile of the PF around the cavity resonance, we express the PF in terms of the Lorentzian LDOS, ρ­(**r**_0_, ω). Using eqs 2 and 3, and noting that *nn*_g_ = *n*_*c*_^2^ for PEC waveguides,^51^ we obtain the SER as

14 $$\Gamma^{C}=\frac{\pi |p{|}^{2}}{\hbar n_{c}^{2}\varepsilon_{0}V_{m}}\omega \rho \left(r_{0},\omega \right)$$

By normalizing this expression to the SER in vacuum and using the Lorentzian form of ρ­(**r**_0_, ω), we arrive at

15 $$F_{P}^{C}=\frac{6\pi }{n_{c}^{2}V_{m}}\cdot \frac{c^{3}}{\omega^{2}}\cdot \frac{\delta \omega }{4{\left(\omega -\omega_{0}\right)}^{2}+\delta \omega^{2}}$$

Using the relations δω = ω_0_/*Q*_disp_, *Q*_disp_ = *Qn*_g_ = *Qn*_*c*_^2^/*n*, $Q=\left(2n_{c}L_{z}/\lambda_{0}\right)F_{R}$ , $F_{R}=\pi \sqrt{R}/\left(1-R\right)$ , *R* = ((*n* – 1)/(*n* + 1))^2^ and *n* = λ_0_/2*L*_*z*_ along with eq 8, we calculated the PF spectrum near the cutoff wavelength λ_*c*_ = 1 μm. The result is shown in Figure 2 (dashed curve) for an open-ended vacuum-filled PEC cavity with dimensions *L*_*x*_ = 0.5 μm, *L*_*y*_ = 1 μm, and *L*_*z*_ = 20 μm, and a confinement factor of α = 0.25 (SI, Section 3), corresponding to the dominant TE_101_-like mode. The plot reveals that, due to the finite length of the cavity, the resonance exhibits a blue shift relative to the PF peak of the infinite waveguide case (solid curve).

### 2.2 Purcell Factor in ENZ BR Microcavities

Before analyzing the PF in an ENZ BR microcavity, as illustrated in Figure 1a, it is important to highlight two key differences between waveguides or cavities with PEC or metallic boundaries and those composed of Bragg reflector components, specifically regarding their ENZ mode profiles and effective mode volumes.

First, in a PEC or metallic waveguide, the dominant TE_10_ mode exhibits an antinode along the *x*-direction, while the field is uniform along the *y*-axis. This allows the waveguide dimension along *y* to be reduced to well below the free-space wavelength without inhibiting the propagation of the TE_10_ mode. In contrast, BRWs with finite width (*L*_*y*_) support fundamental modes that have field antinodes along both *x* and *y*, resembling the fundamental HE_11_ mode in rectangular dielectric waveguides. In these structures, operating near their cutoff wavelength, not only is it impossible to reduce the waveguide width to well below the free-space wavelength, but the width must actually be significantly larger. As discussed in Section 3.2, near cutoff, the effective wavelength is stretched by a factor of 1/*n*, i.e., λ/*n*, and therefore the waveguide width cannot be much smaller than this stretched wavelength. Reducing the width significantly below this value leads to increased propagation losses. As we will demonstrate in this section, this requirement for increased waveguide width, compared to PEC/metallic waveguides, causes the Purcell enhancement to scale as 1/*n*, in contrast to the 1/*n* ^2^ scaling observed in metallic waveguides.

The second difference is related to the effective mode volume of the cavities. In the case of PEC/metallic cavity with homogeneous filling material, effective mode volume is given by *V*_*m*_ = *αV* with the confinement factor α = ⟨|**E**_*m*_(**r**)|^2^⟩/|**E**_*m*_(**r**_max_)|^2^, leading α = 0.25, corresponding to the dominant TE_101_-like mode of the cavity (SI, Section 2).

In a BR cavity, the permittivity profile is homogeneous along the *y*- and *z*-directions but varies inhomogeneously along the *x*-axis. As discussed in SI, Section 4, the electric field distribution along the *y*- and *z*-axes closely follows a sinusoidal profile. Therefore, eq 11 for the effective mode volume can be written as *V*_*m*_ ≈ 0.25 β*V*, where *V* = *L*_*x*_ *L*_*y*_ *L*_*z*_, *L*_*x*_ denotes the core thickness (along the *x*-axis), and β is a confinement factor that captures the influence of the spatially varying permittivity along *x*

16 $$\beta =\frac{1}{L_{x}}\frac{\int_{x}\varepsilon \left(x\right){\left|E_{\mathrm{my}}\left(x\right)\right|}^{2}\mathrm{dx}}{\varepsilon \left(x_{\max}\right){\left|E_{\mathrm{my}}\left(x_{\max}\right)\right|}^{2}}$$

which depends on both the geometry and the permittivity of layers along the *x*-axis.

To analyze how eq 10 scales with the NZI parameter *n* in a typical BR cavity, we use eq 10 and set *L*_*z*_ = λ_0_/2*n*, *L*_*y*_ = λ_0_/4*n* and *L*_*x*_ = λ_c_/2*n*_c_, where λ_c_ is the cutoff wavelength and *n*_c_ is the real part of the core refractive index. Therefore, by using *V*_*m*_ ≈ 0.25 β*V* and eq 16, the PF is obtained as *F*_P_^BRC^ ≈ (48/π^2^β) *nn*_c_ *Q*. Substituting $Q=\left(2n_{c}L_{z}/\lambda_{0}\right)F_{R}\approx \pi n_{c}/4n^{2}$ for near-zero values of *n*, and incorporating local-field effects, yields

17 $$F_{P}^{\mathrm{BRC}}\approx \frac{12L^{2}}{\pi \beta }\frac{n_{c}^{2}}{n}$$

where *L* is the Lorentz–Lorenz local-field correction factor, *L* = (2 + *ε*)/3 for an atom inside the core.^46,52^ In the ENZ regime where the effective epsilon tends to zero (*ε* → 0), this factor reduces to *L* ≈ 2/3.

## 3 Results and Discussion: Numerical Analysis

In this section, we present numerical results and discussion of the Purcell effect in ENZ microcavities. The PF is defined as the ratio of the LDOS, ρ­(**r**_0_, ω), at the emitter position in a structured environment to the free-space LDOS, ρ_0_(ω). The LDOS for a dipole oriented along the unit vector **û**, located at **r**_0_, is proportional to the imaginary part of the electric dyadic Green’s function evaluated at coinciding source and observation points, **G** (**r**_0_, **r**_0_; ω). Specifically, the PF is given by

18 FP=ρ(r0,ω)ρ0(ω)=û·Im{G(r0,r0;ω)}·ûû·Im{G0(r0,r0;ω)}·û

where **G** and **G**_0_ denote the electric dyadic Green’s functions in the structured and free-space environments, respectively. This formulation inherently captures both the spatial and polarization dependence of the emitter-field interaction.

To numerically implement this formalism, we perform COMSOL simulations that exploit the mathematical analogy between quantum and classical descriptions of spontaneous emission. According to Poynting’s theorem, the radiated power of any current distribution **j**(**r**) with harmonic time dependence in a linear medium equals the rate of energy dissipation, given by

19 dWdt=−12∫VRe{j*(r)·E(r)}dV

For a current distribution corresponding to a point dipole emitter, **j**(**r**) = −*i*ω**p**δ­(**r** – **r**_0_), with dipole moment **p** located at **r**_0_, the dissipated power can be rewritten in terms of the Green’s dyadic function as

20 dWdt=ω32c2ε0εIm{p·G(r0,r0;ω)·p}

establishing a direct connection between quantum and classical formalisms for Purcell factor evaluation.^50^ Following this approach, we place a gold nanoparticle carrying a harmonic current density, effectively acting as an oscillating dipole emitter, at the center of the cavity. We then compute the dissipated power and normalize it to the corresponding value in free space, enabling us to determine the PF within the structure.^53^

### 3.1 Numerical Modeling of Waveguides and Cavities with PEC or Metallic Boundaries

In practice, the divergence of PF at zero effective index of refraction, as predicted by eqs 5 and 10, is prevented by material losses. The detrimental impact of material losses on PF is much more pronounced in resonant cavities due to multiple passes of light inside the cavity. To demonstrate this effect, the calculated PF curves for cavities formed by either PEC (solid curve) or gold (dashed curve) walls are shown in Figure 3a. The wavelength-dependent optical constants of gold are taken from the measurements reported by Johnson and Christy.^54^ The cavity dimensions are *L*_*x*_ = 0.5 μm, *L*_*y*_ = 1 μm, and *L*_*z*_ = 8 μm (Figure 3b). The dipole moment of the emitter is along *y*-axis, exciting the lowest order TE_10_ mode of the cavity. For the case of gold cavity, *L*_*x*_ is reduced by the value of skin depth to keep the cutoff at λ_c_ = 1 μm. The resonance feedback is provided by impedance mismatch through inclusion of two waveguide sections filled with materials of refractive index *n* = 2 at the cavity ports.

**Figure 3:**
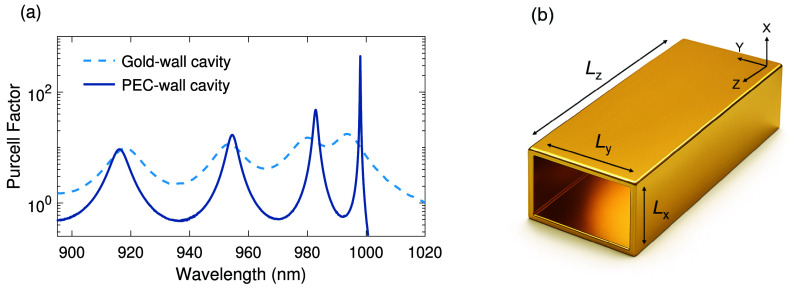
(a) Numerically calculated PFs for cavities with PEC (solid curve) and gold (dashed curve) walls and cutoff wavelength of λ_c_ = 1 μm. (b) Schematic view of the gold-walled cavity.

The curves show that the free spectral range and spectral width of the resonances are reduced in both PEC and gold-wall resonators, when approaching the cutoff wavelength. This is due to increased dispersion inside the cavities near the cutoff wavelength. However, the *Q* and PF of the gold cavity are considerably lower than those of the PEC cavity, due to the ohmic losses of gold. The losses also adversely affect the dispersion profile of modes in a waveguide. As depicted in Figure 4a, the dispersion of the real part of effective index *n*, and consequently *n*_g_ in a PEC waveguide tends to infinity when approaching the cutoff wavelength of λ_*c*_ = 1 μm. However, for a gold-wall waveguide (Figure 4b), the group index is maximized already before the cutoff and gradually tends to zero at longer wavelengths accompanied by a substantial rise in imaginary part of effective index κ, responsible for propagation losses.

**Figure 4:**
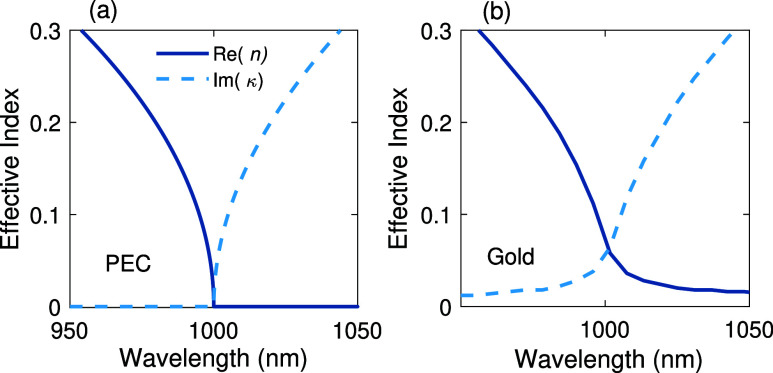
Dispersion curves of (a) a PEC waveguide, (b) a gold-wall waveguide. The transverse dimensions of the PEC and gold wall waveguides are the same as in Figure 3.

Three-dimensional (3D) COMSOL simulations were also conducted to study the dependence of PF and *Q* (blue dots in Figure 5a,5b) on the effective index of refraction (*n* = λ_0_/2*L*_*z*_) in a PEC-wall cavity, through changing the cavity length *L*_*z*_. The cavity core and its surroundings are vacuum, with resonance feedback arising from reflections at the cavity ports due to impedance mismatch. The straight lines in Figure 5, correspond to different functional dependence on 1/*n*. The results confirm the scaling of PF and *Q* as *n* ^–2^ and *n* ^–3^, respectively.

**Figure 5:**
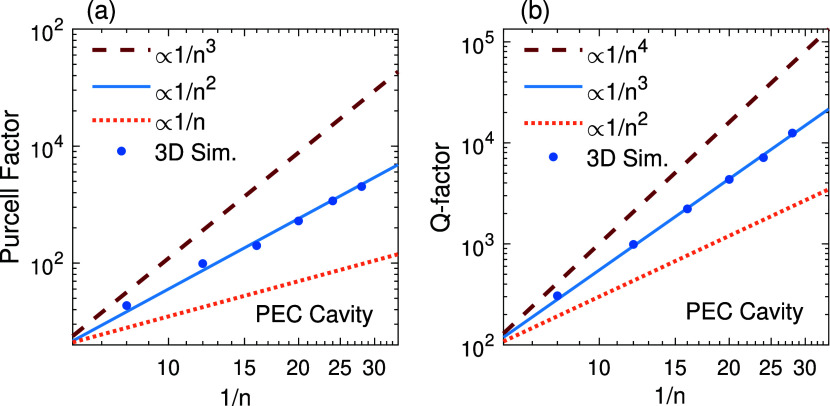
Simulation results (blue dots) showing (a) PF and (b) *Q* values in PEC-wall cavities with *L*_*x*_ = 0.5 μm, *L*_*y*_ = 1 μm, and *L*_*z*_ = 4, 6, 8, 10, 12, 14 μm, corresponding to 1/*n* ≈ 8, 12, 16, 20, 24 and 28, respectively. The lines represent different dependence of PF and *Q* on 1/*n*.

To compare the computed PF values at the center of the cavity with those predicted by eq 12, the parameter α is set to 0.25, corresponding to the dominant mode of the cavity (SI, Section 2). The simulation results for the PF and *Q* are nearly 1.5 times higher than those predicted by eqs 12 and 13. This discrepancy can be attributed to the inaccuracy of the reflectivity expression used at the cavity ports, given by *R* = ((*n* – 1)/(*n* + 1))^2^. While this relation holds for an infinitely extended boundary, the finite size of the cavity ports, comparable to the wavelength, introduces diffraction effects that deviate from this idealized model.^55,56^ Even a small variation in reflectivity can lead to significant changes in the *Q* and consequently the PF. For example, based on the relation $F_{R}=\pi \sqrt{R}/\left(1-R\right)$ , increasing the mirror reflectivity from *R* = 90% by just 3.3% leads to nearly a 50% enhancement in the cavity quality factor *Q*.

### 3.2 Numerical Modeling of All-Dielectric BR Waveguides

The guided waves in BRW structures propagate parallel to the Bragg walls with very little losses compared to common ENZ structures. The effective index of refraction is given by eq 6,^57^ where *n*_*c*_ represents the refractive index of the BRW core material and *L*_*x*_ as the waveguide core thickness. In principle, the radiative losses through the Bragg walls can be arbitrarily reduced by increasing the number of cladding layers. Therefore, the supported low-loss ENZ mode shows strong dispersion and large group index near the cutoff frequency^58^ (Figure 6a), behaving thus very similar to PEC waveguides (Figure 4a). The magnified view of the dispersion curve near the cutoff wavelength, is shown in Figure 6b in a logarithmic scale. The dispersion curves are obtained by two-dimensional (2D) COMSOL mode-analysis simulation of a planar quarter-wave BRW with infinite lateral dimensions. The electric field polarization of the mode is parallel to the Bragg reflectors. The waveguide core material is assumed to be borosilicate glass while the cladding structures on each side consisted of 20 alternating pairs of borosilicate and TiO_2_ layers, with phase thicknesses of π/2 and refractive indices of *n*_BK7_ = 1.51 + i9.93 × 10^–9^ ^59^ and *n*_TiO_2__ = 2.31 + i10^–6^.^60^ The magnified view of the BRW dispersion curve in Figure 6b shows that the FoM of the waveguide at λ = 999.5 nm is as high as *ε*_r_/*ε*_i_ = *n*_2_ – κ_2_/2*nκ* ≈ 2677, with *n* = 0.053 and κ = 9.9 × 10^–6^ as the real and imaginary parts of the effective index.

**Figure 6:**
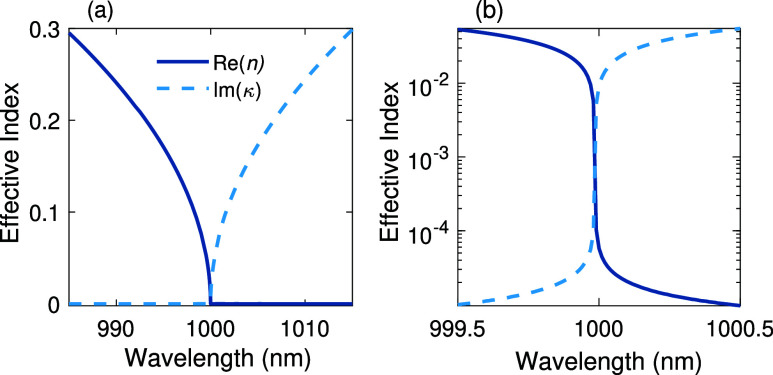
Dispersion curves of (a) a BRW around the cutoff wavelength of λ_c_ = 1 μm. (b) A magnified view of the BRW dispersion curve near the cutoff wavelength, shown in logarithmic scale.

In a half-wave core BRW with virtually infinite lateral dimensions, a continuous spectrum of propagating modes exists for frequencies below the cutoff. These modes can be excited by illuminating the structure at different angles of incidence (with respect to the *x*-axis in Figure 1a). These propagating modes also serve as the resonant modes of the structure, effectively forming a half-wave Fabry–Pérot cavity along the *x*-axis. The resonances are spectrally located within the bandgap of the Bragg mirrors.

Illumination at normal incidence primarily excites the resonance closest to the cutoff, with the corresponding wavelength given by eq 8 in the limit *L*_*z*_ → ∞. As the angle of incidence increases, other resonant modes at shorter wavelengths can be excited. These resonances manifest as sharp transmission peaks within the bandgap of the Bragg mirrors, as shown in Figure 7a and more clearly in the magnified view in Figure 7b, for different numbers of cladding layers. The resonant transmission peak blue-shifts from the cutoff wavelength (Figure 7c) as the angle of incidence increases, reflecting the excitation of shorter-wavelength ENZ modes.

**Figure 7:**
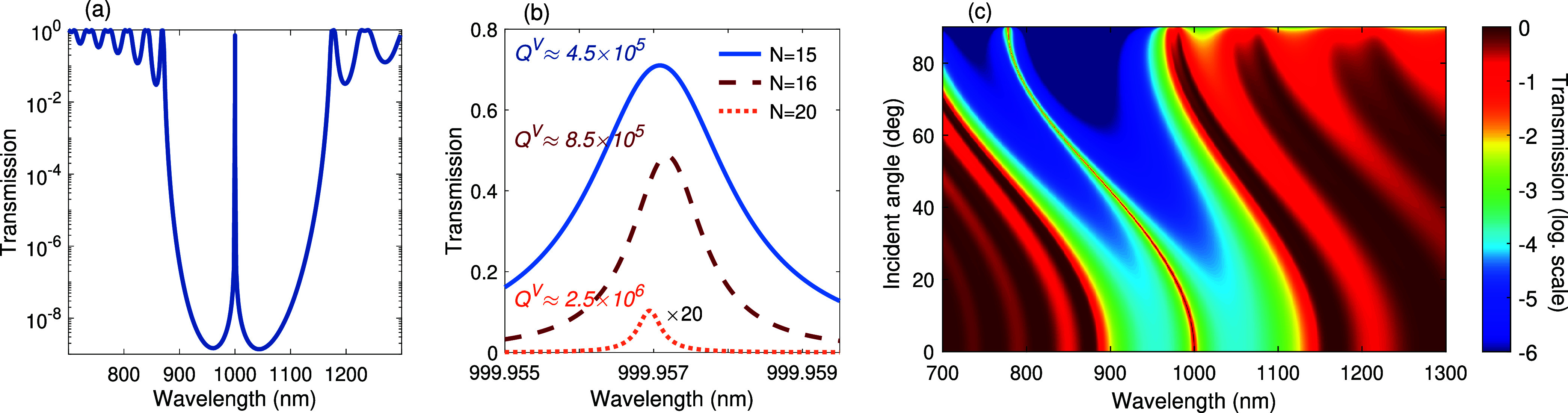
Transmittance through the BRW with parameters as in Figure 6a, with electric field polarization parallel to the Bragg layers. (a) Normal incidence transmittance corresponding to the number of cladding pairs of *N* = 15. (b) A zoomed view of normal incidence transmission peaks for *N* = 15, 16 and 20, represented by solid, dashed and dotted curves, respectively. (c) Modification of transmittance profile and resonance shift as a function of incidence angle.

In contrast to infinitely extended BRWs, truncated BRWs operating near the cutoff wavelength, exhibit strong reflections from the waveguide terminations, leading to cavity-like behavior along the horizontal resonator axis. As a result, only a discrete set of BRW modes can propagate and resonate along the cavity axis, in a condition specified by eq 7. Coupling light into such near-cutoff cavities through the ports is challenging due to significant impedance mismatch with the surroundings. Instead, illumination near normal incidence provides an effective means of exciting the cavity ENZ modes, with each mode corresponding to a specific cavity length (eq 8) and incidence angle of the incoming wave.

While the excited cavity modes may lie very close to or far from cutoff, the dispersion curves in Figure 6b indicate that the high-FoM ENZ regime is restricted to a rather narrow spectral window near the cutoff wavelength. This behavior arises because, as the cutoff wavelength is approached, while the real part of the effective index decreases rapidly, the imaginary component increases concurrently, leading to lower FoMs. When the waveguide is truncated along the *z*-axis to support a cavity resonance, the fundamental resonance condition *L*_*z*_ = λ_0_/2*n* implies that a decreasing effective index near cutoff necessitates longer cavity lengths to sustain the fundamental mode. However, the high-FoM condition imposes an upper limit on the cavity length. For instance, according to Figure 6b, the effective index value of *n* = 0.05, corresponds to the FoM of *n*/κ = 2677 (and the cavity length of *L*_*z*_ = λ_0_/2*n* ≈ 10 μm). As *n* decreases to 0.005 with κ = 10^–4^, the cavity length increases to *L*_*z*_ ≈ 100 μ m, while the FoM drops to 50. At exact cutoff, where *n* = κ = 0.001, the cavity length reaches *L*_*z*_ = 500 μm, and the FoM declines to unity. These results suggest that, in realistic scenarios, high-FoM ENZ Bragg cavities are inherently limited to lengths on the order of a few hundred micrometers or less.

Moreover, maintaining high FoMs while achieving near-zero effective indices is also constrained by the cavity width (*L*_*y*_). Our mode analysis simulations indicate that reducing the cavity width increases lateral radiative losses, thereby lowering the FoM. Conversely, increasing *L*_*y*_ mitigates radiative losses along the *y*-axis (SI, Section 3). Specifically, for a BRW with the same parameters as in Figure 6a, but with limited width of *L*_*y*_ = 10 μm, simulation yields an effective index of *n* = 0.047 + i1.29 × 10^–5^, at λ = 999.5 nm, corresponding to FoM of 1822. Expanding the width to 15 μm (20 μm), raises the FoM to 2483 (2619), approaching the upper limit of 2677 defined in Figure 6a for an infinitely wide waveguide (modeled using periodic boundary conditions on the lateral boundaries). This means that the dispersion curves of laterally infinite BRWs set the upper bound for the FoM in BR cavities. Thus, as the cavity length increases to achieve effective indices closer to zero, preserving the waveguide’s FoM requires a proportional increase in *L*_*y*_ relative to *L*_*z*_. This behavior contrasts with PEC structures, where the effective index can be reduced arbitrarily by extending the cavity length, resulting in a fundamentally different scaling of the Purcell factor with respect to 1/*n* in BR cavities compared to PEC cavities.

### 3.3 Numerical Modeling of All-Dielectric BR Microcavities

To validate eq 17 numerically, full-wave 3D simulation of a BR cavity shown in Figure 1a is required. However, COMSOL simulation of such structures with dimensions much larger than the wavelength resulted in significant convergence issues. These challenges arose from the large simulation domain and the high number of quarter-wave Bragg layers, which demanded an extremely fine mesh. To overcome this and study the dependence of *Q*/PF on the near-zero (NZ) cavity index, we replaced the BRs in the COMSOL simulations with highly reflective thin-film virtual mirrors (VMs) (SI, Section 4). We assigned the VM with an NZ refractive index to avoid fine-mesh requirements in simulations. To mimic BRs, we also assigned the VM with an NZ-impedance to exhibit high reflectance. Properly tuning the permittivity and permeability of the VM allows its reflectance to match that of real BRs. To further facilitate simulation convergence and reduce computation time, we considered cavities with lower *Q*s, on the order of 10^5^. In the SI, Section 4, it is shown that a typical laterally infinite cavity with SiO_2_ core and Bragg reflectors each composed of 16 pairs of quarter-wave layers exhibits a *Q* of ≈ 2.78 × 10^5^ along *x*-axis. By employing a 40 nm thin-film VM with permittivity *ε*_VM_ = 3.65 × 10^3^ and permeability μ_VM_ = 10^–5^, we achieved reflectivities closely matching those of the BRs in the spectral range of interest. As demonstrated in SI, a Fabry–Pérot cavity with SiO_2_ core sandwiched between these VMs closely replicates the mode and transmission profiles as well as *Q* of the BR cavity. We computed the PF and *Q* of the same VM cavity but with several finite lengths (*L*_*z*_) and the same size ratio of *L*_*z*_/*L*_*y*_ = 2 used to derive eq 17. The results, expressed in terms of the inverse of the NZ effective index are shown in Figure 8, where the blue dots denote the simulation results. The solid blue line in Figure 8a corresponds to a function proportional to 1/*n*, and the dashed line represents a function with an inverse square dependence on 1/*n*.

**Figure 8:**
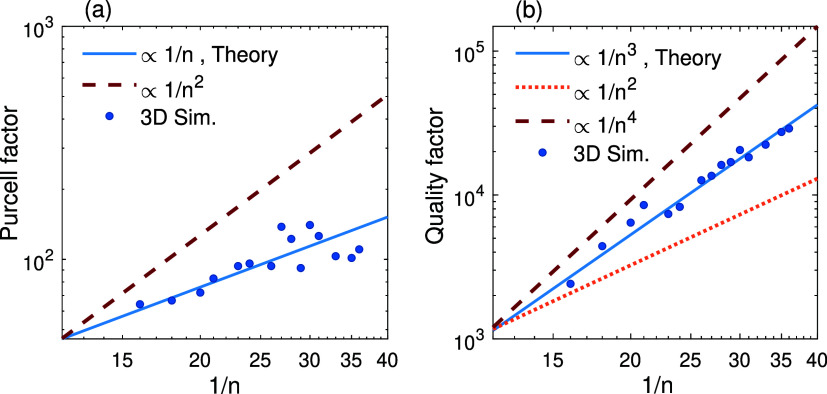
Simulation results (blue dots) showing (a) PF and (b) *Q* values in VM cavities with cutoff wavelength of λ_*c*_ = 1000 nm, *L*_*x*_=λ_*c*_/2*n*_c_ (half-wave thickness), *L*_*y*_=*L*_*z*_/2, and several values of *L*_*z*_ from 8 to 18 μm, corresponding to 1/*n* ≈ 16–36. The lines represent different proportionality of PF and *Q* on 1/*n*.

The cubic dependence of the *Q* on 1/*n*, is clearly confirmed in Figure 8b, similar to the case of PEC cavity. Although the cavity is not lossless (the core refractive index is complex; *n*_*c*_ = 1.4505 + *i*10^–6^), the simulation data in Figure 8a,8b, nearly follow the same tend predicted for lossless cavities, scaling as 1/*n* for the PF and 1/*n* ^3^ for the *Q*.

Using the core index *n*_*c*_ = 1.4504, local-field correction factor *L* = 2/3, and the confinement factor β ≈ 1.53 (SI, Section 2.2), the functional dependence of the PF on *n* is obtained from eq 17 as *F*_P_^BRC^ ≈ 2.33 × *n* ^–1^. However, the proportionality constant in this expression is nearly 1.63 times lower than that of the fitted function (3.8 × *n* ^–1^) shown in Figure 8a. This discrepancy is primarily attributed to the limitations of the idealized reflectivity model used at the cavity ports, which assumes a perfectly planar, infinitely extended interface described by R = ((*n* – 1)/(*n* + 1))^2^ in terms of the NZI, *n*. In reality, the BR cavity features a subwavelength, multilayered termination, which introduces diffraction effects not captured by the simplified model.

While the simulation results in Figure 8a (blue dots) generally follow the 1/*n* trend predicted by theory (solid line), some data scatter is observed around the fitted curve, particularly at higher values of 1/*n*. These deviations are attributed to numerical inaccuracies in COMSOL, which become more pronounced as the cavity size increases with 1/*n*, making convergence more challenging and reducing simulation precision.

To express the PF in terms of an overall cavity *Q*, which accounts for different output coupling mechanisms, we note that there are two primary sources of radiation loss: vertical (V) leakage through the Bragg reflectors along *x*-axis and horizontal (H) leakage through the cavity sidewalls or terminations. Consequently, the overall output coupling *Q* is given by 1/*Q* = 1/*Q* ^V^ + 1/*Q* ^H^ with *Q* ^H^ ≈ π*n*_*c*_/4*n* ^2^. Thus, by using *F*_P_^BRC^ ≈ (48/π^2^β)*nn*_c_ *Q*, the PF can be written as

21 $$F_{P}^{\mathrm{BRC}}\approx \frac{48nn_{c}}{\pi^{2}\beta }\frac{Q^{H}}{1+Q^{H}/Q^{V}}$$

which for *Q* ^V^ ≫ *Q* ^H^, reduces to eq 17, regardless of the local-field correction factor. However, when *Q* ^V^ becomes comparable to *Q* ^H^, deviations from eq 17 emerge.

Therefore, the upper limit of the *Q* for BR cavity is ultimately set by *Q* ^V^, which is primarily constrained by material and radiative losses associated with the Bragg mirrors. These losses can be mitigated by employing low-loss, high-purity core and cladding materials, using superpolished substrates to minimize scattering, and incorporating a large number of cladding layers to suppress radiative leakage. Using such techniques, Bragg cavities with exceptionally high finesse, on the order of 10^5^–10^6^, have been demonstrated.^61,62^ Consequently, in practice, the PF in BR cavities is limited by the feasibility of achieving sufficiently high mirror reflectivity. While commercial dielectric supermirrors with reflectivities exceeding 99.998% are available, microscale structures can exhibit reduced reflectivity and *Q* due to finite lateral size and scattering from etched sidewalls. Nevertheless, very high reflectivities have already been experimentally demonstrated in structures with lateral dimensions ranging from a few micrometers to a few tens of micrometers.^63,64^

## 4 Concluding Remarks

In summary, we have introduced all-dielectric BRWs and microcavities operating near their cutoff frequency as a promising ENZ platform, serving as an ultralow-loss counterpart to metallic or PEC structures. We have derived scaling laws for the *Q* and PF in such lossless or minimally lossy ENZ microcavities. For PEC-walled ENZ rectangular cavities, these factors scale as *n* ^–3^ and *n* ^–2^, respectively, with the near-zero effective refractive index, *n*. In all-dielectric ENZ BR cavities near cutoff, the scaling follows *n* ^–3^ for the *Q* and *n* ^–1^ for the PF. However, by increasing the cavity size, the upper limit of these factors is constrained by the reflectivity of the Bragg mirrors.

Thanks to state-of-the-art dielectric multilayer coating techniques, it is feasible to fabricate mirrors with extremely high reflectivities exceeding *R* = 99.99984%.^62^ Furthermore, the implementation of compact 3D Bragg cavities with laterally confined geometries is enabled by advanced micro- or nanolithographic methods, making these ENZ structures practically realizable within current dielectric or semiconductor manufacturing technologies.^63,64^ It should be noted, however, that increasing the cavity *Q*, while beneficial for enhancing the Purcell effect and energy confinement, also necessitates narrower-bandwidth optical excitation sources to efficiently couple light into the cavity. This trade-off must be carefully considered in the design of high-*Q* ENZ photonic systems.

Despite these challenges, our results indicate that ENZ BR microcavities offer a promising platform for low-threshold lasing, phase-matching-free nonlinear optics, and the investigation of strong light-matter interactions in quantum optics.

## Supplementary Material



## Notes

Discussion of impedance mismatch in cavity terminations and estimation of reflectivity finesse in ENZ microcavities in terms of effective index of refraction; calculation of confinement factors for both PEC and BR cavities; mode-analysis numerical calculations related to BR microcavities; and finally details of implementing thin-film virtual mirrors instead of Bragg mirrors in COMSOL simulations of 3D BR microcavities (PDF)
