# Supplementary material for: Purcell Effect in Epsilon-Near-Zero Microcavities
Source: ACS Omega. 2025 Sep 20;10(38):44683–92. doi: 10.1021/acsomega.5c07448 (PMC12489649; doi:10.1021/acsomega.5c07448)
Supplement: Supplementary file 1 [file ao5c07448_si_001.pdf]

Supporting Information for:

Purcell Effect in Epsilon-Near-Zero Microcavities

Ali Panahpour*
Jussi Kelavuori
Mikko Huttunen

Photonics Laboratory, Physics Unit, Tampere University, FI-33014 Tampere, Finland

*Corresponding author: ali.panahpour@tuni.fi

Abstract

This document provides supplementary information to "Purcell Effect in Epsilon-Near-Zero Microcavities"

1 Impedance mismatch in cavity terminations and reflectivity finesse in ENZ microcavities in terms of effective index of refraction

In Bragg-reflection (BR) or perfect electric conductor (PEC) cavities described in the main text, the resonance feedback along the cavity axis (z direction) is provided by partial reflections from the cavity terminations. The reflections depend on the contrast between the characteristic impedance of the waveguiding structure, $Z_{\mathrm{w}} = \sqrt{\mu/\epsilon}$ in terms of the effective permeability and permittivity of the waveguide and the impedance of the surrounding medium, Z_{s}. The reflection coefficient at the ports is given by [1]:

$$R = \left(\frac{Z_{\mathrm{s}} - Z_{\mathrm{w}}}{Z_s + Z_{\mathrm{w}}}\right)^2 . \quad (1)$$

Using the relations $Z_{\mathrm{s}} = Z_0/n_{\mathrm{s}}$ and $Z_{\mathrm{w}} = Z_0/n$, where the impedances are expressed in terms of the free-space impedance (Z_0) and the effective refractive indices of the waveguide (n) and surrounding medium (n_{s}), the reflectivity simplifies to:

$$R = \left(\frac{n - n_{\mathrm{s}}}{n + n_{\mathrm{s}}}\right)^2 . \quad (2)$$

Thus, in the ENZ regime when n tends to zero, the reflectivity approaches the unity and the output coupling from the cavity ports is strongly influenced by the cavity effective index. Regarding the reflectivity finesse $\mathcal{F}_R = \pi\sqrt{R}/(1 - R)$ of the cavity with symmetrical ports, in the limit of $n \to 0$, applying Eq. (2) and simplifying the finesse expression, we obtain:

$$\mathcal{F}_R \approx \frac{\pi}{4}\frac{n_s}{n} \propto \frac{1}{n} . \quad (3)$$

Our simulation results in the main text (Figs. 5 and 8) confirm the validity of this proportionality of reflectivity finesse to $1/n$ in the calculation of the Purcell and quality factors.

However, the proportionality constant $(\pi/4)n_{\mathrm{s}}$ in Eq. (3) is not perfectly accurate, because while Eq. (2) generally assumes an infinitely ex-

tended boundary, the finite size of the cavity ports—comparable to the wavelength—introduces diffraction effects that cause deviations from this idealized model [2, 3]. Even small variations in reflectivity can lead to significant changes in the quality factor Q, and consequently in the Purcell factor (PF).

Due to the impedance or index mismatch at the terminations of a truncated waveguide, reflections occur at the ports, causing the waveguide to behave like a resonant cavity that supports standing-wave modes. The resonance condition follows the Fabry–Pérot criterion, which requires that the round-trip phase accumulation inside the cavity equals an integer multiple of 2π. As demonstrated in Sec. 2 of this Supplementary Information (SI) document, the dominant mode along the cavity axis in PEC or BR ENZ waveguides closely follows a sinusoidal profile. The large index contrast with the surroundings (e.g., vacuum) causes both interfaces to act like hard boundaries ($n \ll n_s$), each contributing a reflection-induced phase shift of $\approx \pi$. These shifts add up to $\approx 2\pi$, corresponding to a full-cycle phase advance and thus not affecting the resonance condition. As a result, the phase accumulation condition simplifies to the standard form, $nL_z \approx m\lambda/2$ or:

$$n \approx \frac{m\lambda}{2L_z}, \tag{4}$$

where m is an integer. This relation describes the standing-wave modes in a finite-length ENZ waveguide, which effectively behaves as a resonant cavity due to reflections at its terminations. The resonance condition is approximate, since the cavity mode, although nearly sinusoidal, shows slight deviations near the terminations, where the ports do not act as perfectly hard boundaries like ideal PEC interfaces, as illustrated in Figs. S3 and S8 of SI, Sec. 2.

The effective refractive index in a PEC waveguide filled with a material of refractive index n_c is given by [1]

$$n(\lambda) = n_c\sqrt{1 - \left(\frac{\lambda}{\lambda_c}\right)^2}, \tag{5}$$

where $\lambda_c = 2\pi n_c/k_c$ is the cutoff wavelength of the guided mode and $k_c = \pi/L_x$ is the cutoff wavenumber of the dominant TE_{10} mode [1]. Therefore, the resonance wavelength is determined by the approximate condition

$$n_c\sqrt{1 - \left(\frac{\lambda}{\lambda_c}\right)^2} \approx \frac{m\lambda}{2L_z}, \tag{6}$$

where m is the longitudinal mode number.

Equation (5) also applies to a Bragg-reflection waveguide (BRW), where n_c is interpreted as the refractive index of the BRW core material and L_x as the waveguide core thickness [4].

2 Calculation of confinement factors

2.1 PEC cavity

As discussed in the main text, assuming a homogeneous material inside the PEC cavity, the effective mode volume expression in Eq. (11) is reduced to the form

$$V_m = \frac{\int_V |\mathbf{E}_m(\mathbf{r})|^2 \, \mathrm{d}\mathbf{r}}{|\mathbf{E}_m(\mathbf{r}_{\max})|^2}. \tag{7}$$

For the dominant TE_{101}-like cavity mode the electric field along y direction is uniform, while it has a sinusoidal form along x direction [1]. This sinusoidal profile is also shown numerically in Fig. S2 for a PEC cavity shown schematically in Fig. S1 with dimensions $L_x = 500$ nm, $L_y = 1000$ nm and $L_z = 14$ μm.

As shown in Fig. S3, COMSOL simulations indicate that the field profile of the dominant mode along the z-axis also exhibits a nearly sinusoidal form. Therefore, the electric field intensity inside the cavity can be expressed as a product of sinusoidal functions along the x- and z-directions:

$$|\mathbf{E}_m(\mathbf{r})|^2 \approx |\mathbf{E}_m(\mathbf{r}_{\max})|^2 \cdot \sin^2\left(\frac{\pi x}{L_x}\right) \cdot \sin^2\left(\frac{\pi z}{L_z}\right), \tag{8}$$

and Eq. (7) simplifies to $V_m \approx 0.25V$, or equivalently, $\alpha = V_m/V \approx 0.25$.

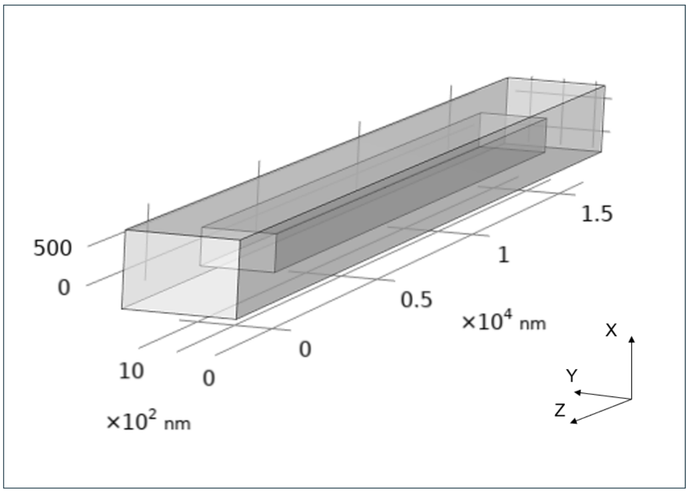

Figure S1: COMSOL simulation domain of a PEC-walled cavity (central rectangular volume) with open ports, embedded in a vacuum environment.

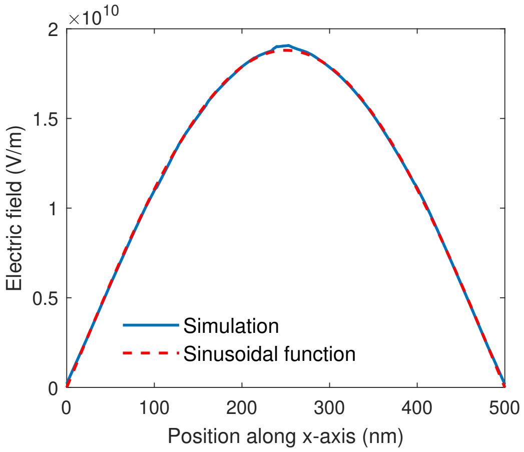

Figure S2: Electric field distribution (solid curve) inside the PEC cavity along the x-axis, compared with a sinusoidal reference profile (dashed curve).

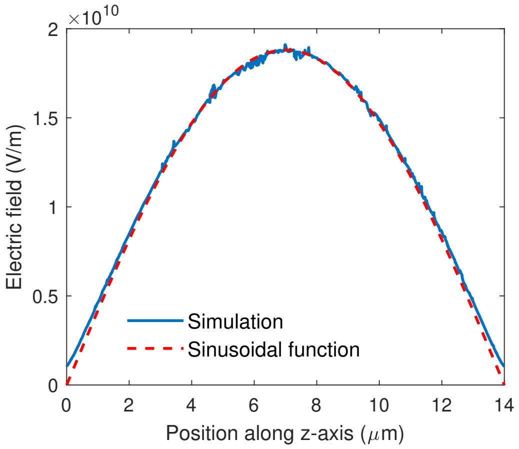

Figure S3: Electric field distribution (solid curve) inside the PEC cavity along the z-axis, compared with a sinusoidal reference profile (dashed curve).

2.2 BR cavity

As discussed in the main text, the permittivity profile of a BR cavity is homogeneous along the y- and z-directions, which lie parallel to the Bragg layers, but varies inhomogeneously along the x-axis, normal to the layers. Figure S4 presents the electric field transverse profile of an ENZ mode, corresponding to the x–y cross-section of a BR cavity with a width of $L_y = 10\ \mu$m. The mode profile is obtained using the 2D mode analysis module of the COMSOL software. The electric field is polarized along the horizontal (y) direction, while the wave vector is oriented normal to the surface (along the z-axis). The cavity comprises a half-wavelength-thick SiO_2 core, sandwiched between two claddings, each consisting of 16 pairs of quarter-wave SiO_2/Ta_2O_5 layers. The real parts of the refractive indices at the cutoff wavelength $\lambda_c = 1000$ nm are $n_{\mathrm{SiO_2}} = 1.4504$ [7] and $n_{\mathrm{Ta_2O_5}} = 2.0990$ [8].

Figure S5 shows the electric field distribution of the mode along the y-direction at the center of the core. The computed mode profile (solid curve) closely matches a sinusoidal function (dashed curve), indicating that the field can be well approximated by such a form. Fig. S6 presents the electric field distribution along the vertical (x-direction) line passing through the center of the BR cavity, as depicted in Fig. S4.

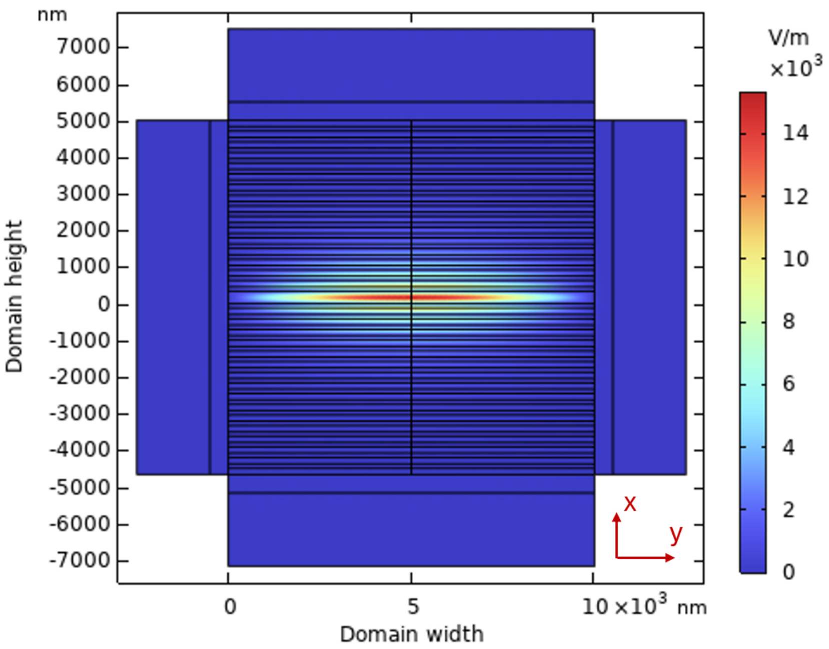

Figure S4: Electric field transverse profile of the dominant ENZ mode across the x–y cross-section of a BR cavity with a width of $L_y = 10\ \mu$m.

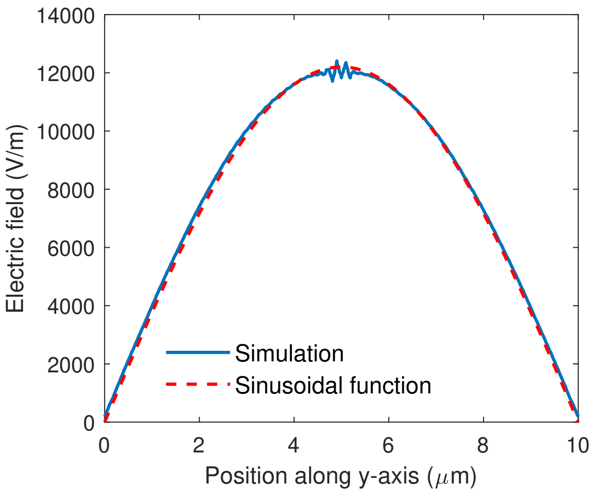

Figure S5: Electric field distribution of the ENZ mode along the y-direction at the center of the core in the BR cavity shown in Fig. S4.

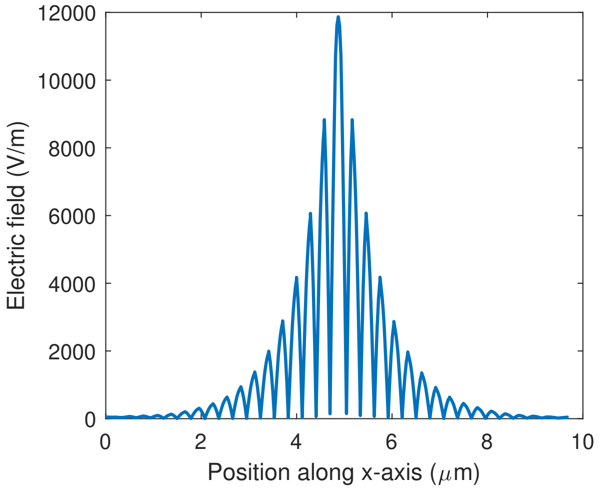

Figure S6: Electric field distribution along the vertical (x-direction) line passing through the center of the BR cavity, depicted in Fig. S4.

Figure S7 shows the longitudinal profile of an ENZ mode in a BR cavity of length $L_z = 20$ μm, along the z-direction. The electric field is polarized out of the simulation plane, while the wavevector is oriented along the z-axis. In this two-dimensional simulation, the ENZ mode is excited inside the cavity by placing a harmonic out-of-plane line current at the center of the core.

The resulting electric field distribution along the z-axis is shown in Fig. S8 (solid curve), which closely matches a sinusoidal profile (dashed curve). Accordingly, the electric field intensity of the mode can be approximated as

$$|\mathbf{E}_m(\mathbf{r})|^2 \approx |E_{my}(x_{\max})|^2 \cdot \sin^2\left(\frac{\pi y}{L_y}\right) \cdot \sin^2\left(\frac{\pi z}{L_z}\right), \tag{9}$$

which, when substituted into Eq. (11) of the main text, yields $V_m = 0.25\,\beta V$, where $V = L_x L_y L_z$ and β is defined as

$$\beta = \frac{1}{L_x} \cdot \frac{\int \varepsilon(x)\,|E_{my}(x)|^2\,dx}{\varepsilon(x_{\max})\,|E_{my}(x_{\max})|^2}, \tag{10}$$

in terms of the inhomogeneous permittivity profile along the x-direction. For the structure shown in Fig. S4, this confinement factor is numerically computed to be $\beta \approx 1.53$.

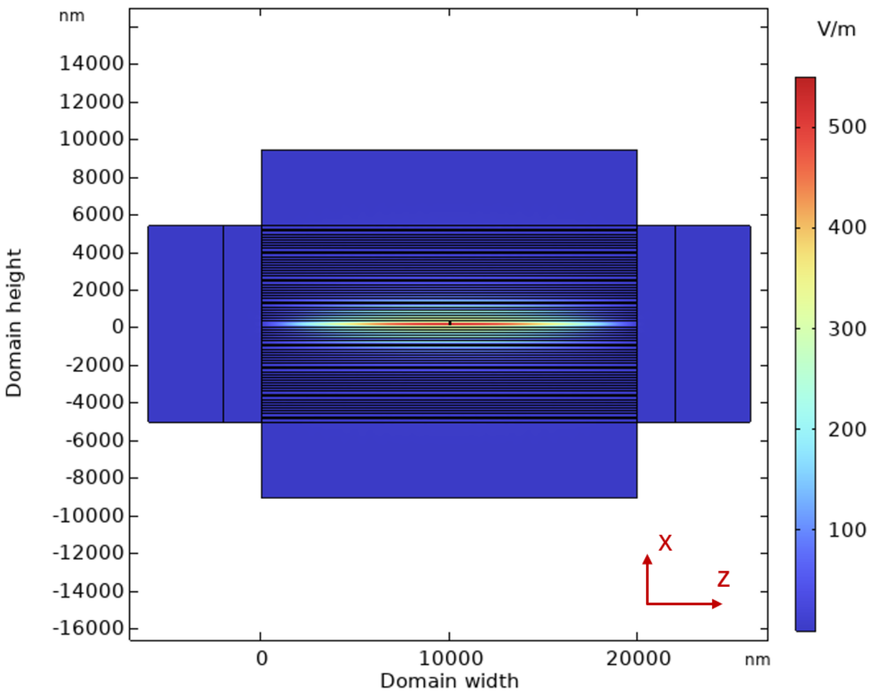

Figure S7: Longitudinal profile of the dominant ENZ mode along the z-direction in a BR cavity of length 20 μm, using the same core and cladding parameters as in Fig. S4.

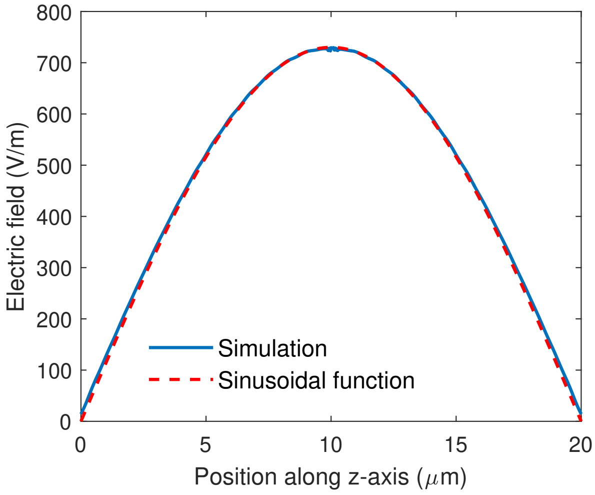

Figure S8: Electric field distribution along the z-axis (solid curve) inside the core of the BR cavity in Fig. S7, which closely matches a sinusoidal profile (dashed curve).

3 Mode analysis results

To calculate the field distribution and effective NZI of the Bragg-reflection waveguide (BRW) modes, we used the 2D Mode Analysis module in COMSOL MULTIPHYSICS. This module performs a wavelength sweep and identifies NZI modes propagating along the z-direction, which is normal to the simulation plane.

Figures S9–S11 show the absolute value of the electric field for the dominant NZI modes of quarter-wave BRWs with widths of 10, 15, and 20 μm, respectively. The cutoff wavelength in all structures is fixed at 1 μm, and the electric field is polarized horizontally, i.e., along the y-axis. The waveguide core is composed of borosilicate glass, while each cladding consists of 20 alternating pairs of borosilicate and TiO_2 layers with optical thicknesses of $\pi/2$. The refractive indices are $n_{\mathrm{BK7}} = 1.51 + \mathrm{i} \times 9.93 \times 10^{-9}$ [5] and $n_{\mathrm{TiO_2}} = 2.31 + \mathrm{i} \times 10^{-6}$ [6].

For the BRW with a width of 10 μm, the effective index of the mode at $\lambda = 999.5$ nm is calculated as $n = 0.047 + \mathrm{i} \times 1.29 \times 10^{-5}$, corresponding to a figure of merit (FoM) of FoM$= n^2 - \kappa^2/2n\kappa = 1822$. As discussed in the main text, increasing the width of the waveguide enhances the FoM, which we attribute to a reduction in radiative losses from the sidewalls. For instance, increasing the width to 15 and 20 μm yields ENZ modes with improved FoMs of 2483 and 2619, respectively, corresponding to effective indices of $n = 0.05529 + \mathrm{i} \times 1.1132 \times 10^{-5}$ and $n = 0.05556 + \mathrm{i} \times 1.0606 \times 10^{-5}$.

This effect can also be demonstrated by comparing the FoMs of BRWs with different widths at a fixed near-zero effective index, as shown in Table 1.

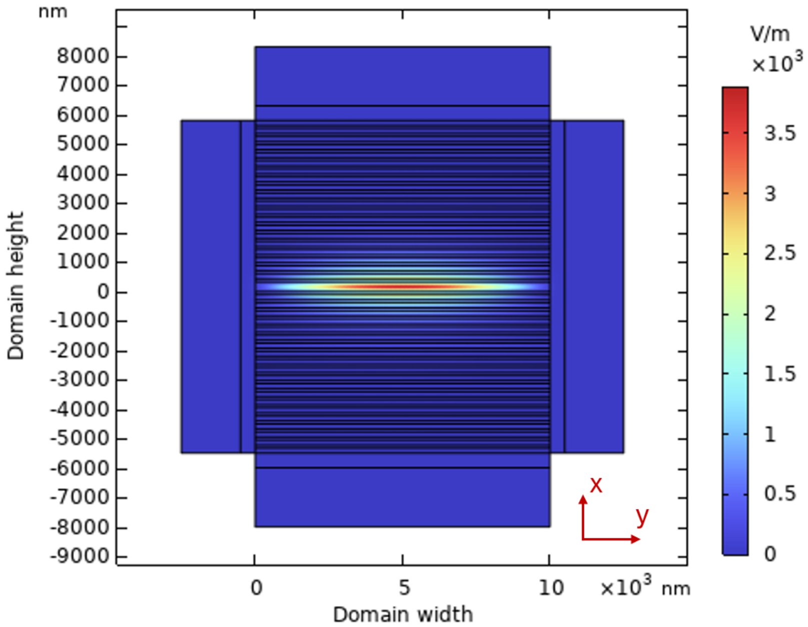

Figure S9: Transverse profile of an ENZ mode in a BRW with the width of 10 μm.

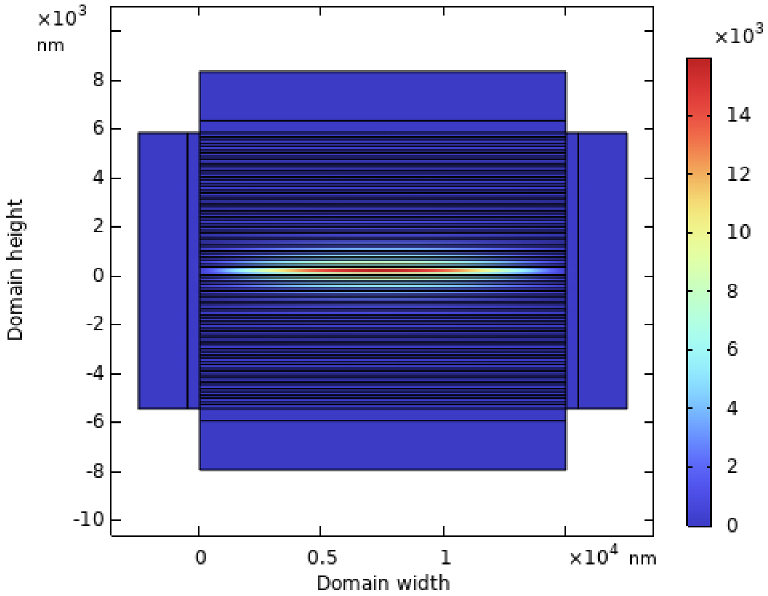

Figure S10: Transverse profile of an ENZ mode in a BRW with the width of 15 μm.

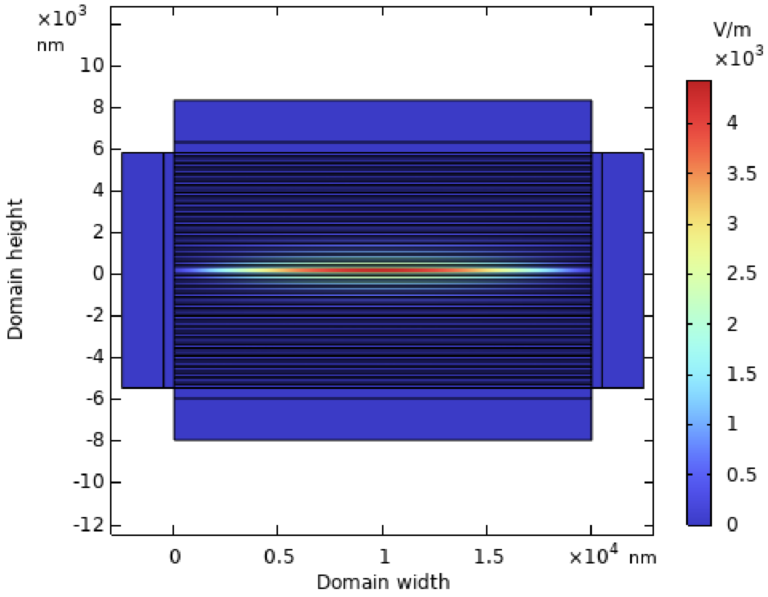

Figure S11: Transverse profile of an ENZ mode in a BRW with the width of 20 µm.

Table S1

NZI	$L_y = 10$ μm	$L_y = 20$ μm
$n \approx 0.036$	$\kappa = 1.6863 \times 10^{-5}$, FoM=1067	$\kappa = 1.6209 \times 10^{-5}$, FoM=1110
$n \approx 0.040$	$\kappa = 1.4980 \times 10^{-5}$, FoM=1335	$\kappa = 1.4657 \times 10^{-5}$, FoM=1364
$n \approx 0.043$	$\kappa = 1.4040 \times 10^{-5}$, FoM=1531	$\kappa = 1.3517 \times 10^{-5}$, FoM=1590
$n \approx 0.047$	$\kappa = 1.2895 \times 10^{-5}$, FoM=1822	$\kappa = 1.2578 \times 10^{-5}$, FoM=1868

Table 1, based on COMSOL mode analysis simulations, shows that increasing the waveguide width reduces the imaginary part of the effective index, thereby enhancing the FoM.

4 Implementing thin-film virtual mirrors instead of Bragg mirrors in COMSOL simulations of 3D Bragg reflector cavities

In this section we compare the performance of a typical BR cavity with a Fabry Perot cavity consisting of specially designed thin film virtual mirrors (VMs). We show that VMs can serve as an effective alternative to BRs

in 3D COMSOL simulations of BR cavities, helping to resolve convergence issues. We consider a BR cavity consisting of a half-wave-thickness SiO_2 core sandwiched between two cladding with 16 pairs of quarter-wave SiO_2 and Ta_2O_5 layers. The materials' refractive indices are $n_{SiO_2} = 1.4504$ [7] and $n_{Ta_2O_5} = 2.0990$ [8] at the cutoff wavelength $\lambda_c = 1000$ nm of the structure. The imaginary part of the refractive indices is assumed to be $\kappa \sim 10^{-6}$. This estimation is based on the experimental data in [9] and reproducing their results by tuning the κ in COMSOL simulation of the same Bragg mirror.

We assume the refractive index of the VMs to be near zero, minimizing fine-mesh requirements in simulations while ensuring high reflectance comparable to Bragg reflectors by assigning them a near-zero impedance. By appropriately selecting the relative permittivity and permeability of the VMs, we can precisely match their reflectance to that of actual BRs.

By employing a 40 nm thin-film VM with permittivity $\varepsilon_{VM} = 3.65 \times 10^3$ and permeability $\mu_{\mathrm{VM}} = 10^{-5}$, we achieve reflectivities closely matching those of the SiO_2/Ta_2O_5 Bragg cavity in the spectral range of interest. Notably, the reflectivity of VMs does not need to be an exact match to that of BRs, as the simulated cavities are relatively small. Consequently, the reflectivity from the cavity ports is significantly lower than that of the BRs, making the Purcell factor or Q-factor primarily dependent on the ports' reflectivity rather than the BR reflectivities.

Considering a half-wave SiO_2 core between the two VMs and illuminating the cavity with a normally incident and horizontally polarized wave, we obtain the mode profile shown in Fig. S12a, which closely resembles that of the BR cavity in Fig. S12b.

The transmission profiles of the VM and BR cavities are also shown in Figs. S13a and S13b, respectively. The similarity in cavity mode and transmission profiles justifies replacing BRs with VMs to mitigate convergence issues.

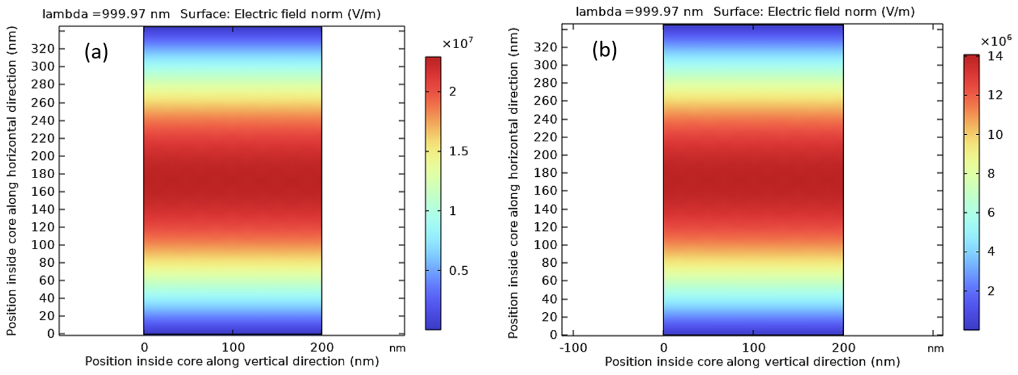

Figure S12: Mode profiles inside the cores of the (a) VM cavity and (b) BR cavity.

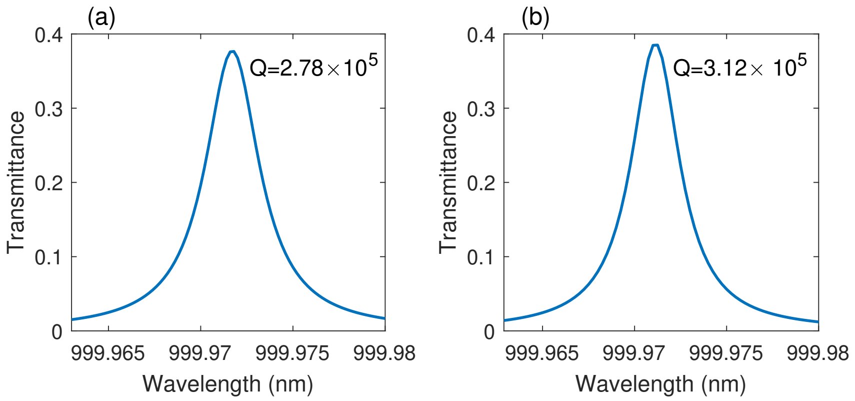

Figure S13: Spectral profiles of the transmission peaks through the (a) VM cavity and (b) BR cavity.

5 References

[1] Pozar, D. M. Microwave Engineering, 4th ed.; John Wiley and Sons, 2011.
[2] Yee, H. Y., L. B. Felsen, and J. B. Keller. "Ray theory of reflection from the open end of a waveguide." SIAM Journal on applied mathematics 16, no. 2 (1968): 268-300.
[3] Li, Lutong, Pu Tang, Bo Chen, Pingyou Wang, and Senhang He. "An approximate method to calculate the reflection coefficient of a rectangular open-ended waveguide." In 2016 Progress in Electromagnetic Research Symposium (PIERS), pp. 1648-1652. IEEE, 2016.

[4] West, Brian R., and Amr S. Helmy. "Properties of the quarter-wave Bragg reflection waveguide: theory." Journal of the Optical Society of America B 23, no. 6 (2006): 1207-1220.
[5] SCHOTT. http://www.schott.com, 2023; Accessed: November, 2023
[6] Zhukovsky, S. V.; Andryieuski, A.; Takayama, O.; Shkondin, E.; Malureanu, R.; Jensen, F.; Lavrinenko, A. V. Experimental demonstration of effective medium approx- imation breakdown in deeply subwavelength all-dielectric multilayers. Physical Review Letters 2015, 115, 177402.
[7] Malitson, Ian H. "Interspecimen comparison of the refractive index of fused silica." Journal of the optical society of America 55, no. 10 (1965): 1205-1209.
[8] Gao, Lihong, Fabien Lemarchand, and Michel Lequime. "Exploitation of multiple incidences spectrometric measurements for thin film reverse engineering." Optics express 20, no. 14 (2012): 15734-15751.
[9] Hagedorn, H., and J. Pistner. "High reflecting dielectric mirror coatings deposited with plasma assisted reactive magnetron sputtering." In Optical Systems Design 2015: Advances in Optical Thin Films V, vol. 9627, pp. 193-198. SPIE, 2015.
